# Hot electron energy relaxation time in vanadium nitride superconducting film structures under THz and IR radiation

**DOI:** 10.1038/s41598-020-73850-2

**Published:** 2020-10-08

**Authors:** Ivan Pentin, Yury Vakhtomin, Vitaly Seleznev, Konstantin Smirnov

**Affiliations:** 1 Moscow State Pedagogical University, Malaya Pirogovskaya Str. 1, Moscow, 119991 Russia; 2 Superconducting Nanotechnology, LLC 5/1-14 L’va Tolstogo Str., Moscow, 119021 Russia; 3 National Research University Higher School of Economics, 20 Myasnitskaya Str., Moscow, 101000 Russia

**Keywords:** Superconducting devices, Sensors

## Abstract

The paper presents the experimental results of studying the dynamics of electron energy relaxation in structures made of thin (d ≈ 6 nm) disordered superconducting vanadium nitride (VN) films converted to a resistive state by high-frequency radiation and transport current. Under conditions of quasi-equilibrium superconductivity and temperature range close to critical (~ T_c_), a direct measurement of the energy relaxation time of electrons by the beats method arising from two monochromatic sources with close frequencies radiation in sub-THz region (ω ≈ 0.140 THz) and sources in the IR region (ω ≈ 193 THz) was conducted. The measured time of energy relaxation of electrons in the studied VN structures upon heating of THz and IR radiation completely coincided and amounted to (2.6–2.7) ns. The studied response of VN structures to IR (ω ≈ 193 THz) picosecond laser pulses also allowed us to estimate the energy relaxation time in VN structures, which was ~ 2.8 ns and is in good agreement with the result obtained by the mixing method. Also, we present the experimentally measured volt-watt responsivity *(S*_~_*)* within the frequency range ω ≈ (0.3–6) THz VN HEB detector. The estimated values of noise equivalent power (*NEP*) for VN HEB and its minimum energy level (δ*E*) reached *NEP*_*@1MHz*_ ≈ 6.3 × 10^–14^ W/√Hz and δ*E* ≈ 8.1 × 10^–18^ J, respectively.

## Introduction

In modern low-temperature physics, the effects of non-equilibrium superconductivity occurring in superconductors in a resistive state are actively studied. In addition to the theoretical interest in the study of such effects, these works also stimulated by applied tasks aimed primarily at the development of receivers for various spectral ranges. For example, the detection and study of the effect of electronic heating in superconducting NbN films, arising under the influence of radiation of different frequencies from the visible to the millimeter wave range, has led to the creation of a new class of cryogenic electronic devices^1,2^. One of these devices, of course, become superconducting NbN HEB (Hot-Electron Bolometer) detectors that exhibit high sensitivity (NEP ≈ 2 × 10^–13^ W/√Hz) combined with record response time (τ ≈ 50 ps), as well as energy resolution δE ≈ 3 × 10^–18^ J^3–5^. Such unique characteristics of NbN HEB detectors made possible by the use of ultra-thin (d ≈ 3–5 nm) disordered NbN films, which are characterized by a low diffusion coefficient (D ≈ 0.5 cм^2^/c), high critical temperature (T_c_ ≈ 10 К), small width of the superconducting transition (∆T_c_ < 0.2 К), the strong temperature dependence of the resistance in the region of the superconducting transition. In addition to NbN films, when studying the effects of non-equilibrium superconductivity and developing receiving devices, nitrides of other transition metals are often used. For example, in the works^7^ the results of studies of thin TiN films having lower operating temperatures compared to NbN were demonstrated and the possibilities of creating detectors based on them were show.

This work presents the results of studies of the energy relaxation times in the vanadium nitride (VN) thin films and the characteristics of the HEB detectors based on them for the first time. The choice of the VN film as the sensitive material of the HEB detectors is due to several factors. First, VN is a technological material, the films of which, like many other nitrides, can be obtained by a fairly simple method of reactive magnetron sputtering of a single-element target^8^. Secondly, the advantage of VN material is its critical temperature (T_c_ ~ 9 K)^9^,which, on the one hand, does not require complex cryogenic equipment as for TiN, and, on the other hand, is somewhat lower than the critical temperature of the NbN or the NbTiN films, which can also be considered as an additional positive property when creating superconducting detectors, for example, single photon detectors in the middle infrared range^10,11^. Third, measured in work^12^ the diffusion coefficient in the VN thin films is close to the diffusion coefficient in the NbN films, which confirmed the possibility of creating the VN detectors at the initial stage of the present work.

## Deposition of the VN films, creation of the VN structures and DC tests fulfillment

VN films were obtained by reactive magnetron sputtering of a vanadium target with a purity of 99.98%. The material was deposited at the Orion series AJA International Inc. facility with typical residual pressure in the vacuum chamber ~ 50 nTorr. The films were deposited on highly resistive silicon substrates (ρ ≥ 10 kOhms × cm), heated to the temperature ~ (700–800) K. The process of reactive magnetron sputtering occurred in the mode of stabilization of the direct current of the magnetron at N_2_ + Ar mixture pressure in the chamber 2.8 mTorr. The deposition rate of the VN films was ~ 0.5 Å/c. Detail description of the VN deposition presented in^13^. The thickness of the created VN films was ~ 6 nm and their surface resistance ~ 100 Ohms/□. The critical temperature value of the obtained films varied within (6.0–6.2) K, which may be due to some small inaccuracies in working gas flows (N_2_ and Ar) in the deposition chamber and the temperature of the substrate holder.

Based on such VN films, two types of structures were created: the superconducting detectors with a sensor element size of 0.4 μm × 0.4 μm, coupled to a spiral logarithmic antenna and without antenna structures with a sensitive region of the film 10 μm × 10 μm. Structures with different sizes of the sensitive region were fabricated to study the effect of electron diffusion on the energy relaxation time of electron temperature. The spiral antenna geometry was optimized for the frequency range (0.3–6) THz in which the volt-watt responsivity measurements were performed. Fabrication process of both structures included electron-beam and photo-lithography, plasma-chemical etching and was similar to fabrication of the NbN HEB^3^.

**Figure 1:**
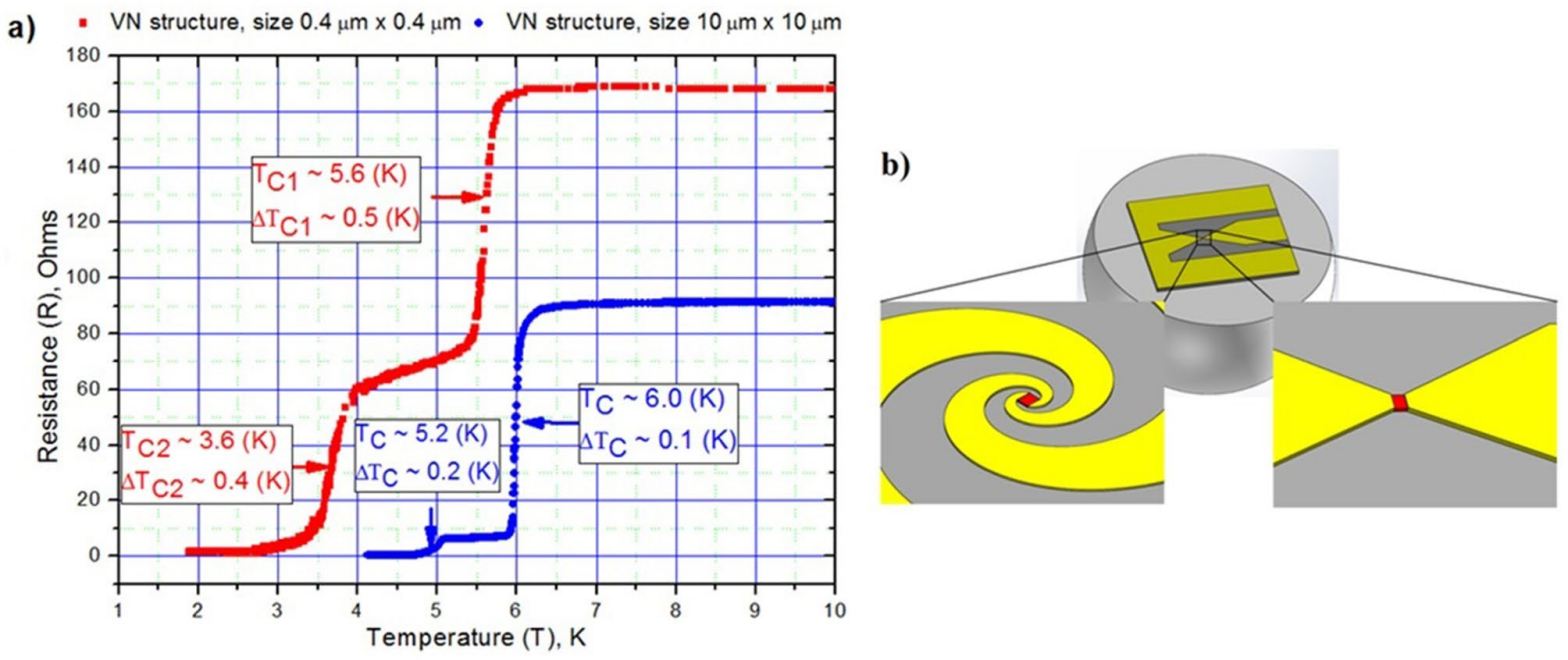
(**a**) The temperature dependences: the VN structure with the size of the sensitive region of 0.4 μm × 0.4 μm (red square), the VN structure with the size of the sensitive region of 10 μm × 10 μm (blue sircle). The graph also shows values of critical temperature (T_c_), the transition width (ΔT_c_). (**b**) Image of a chip of the studied VN structure on a Si substrate, centered at the focus of a hyper-hemispherical Si lens. The sensitive area is a red square. The sensitive area having 0.4 μm (L) × 0.4 μm (W) coupled with a spiral logarithmic antenna (left). The sensitive area 10 μm (L) × 10 μm (W) (right).

The main DC parameters for the VN films and structures created on their basis were: critical temperature of the superconducting transition (T_c_), the superconducting transition width (ΔT_c_), the structure resistance at 300 K (R_300_), metallicity, i.e. RRR = R_300_/R_20_ ratio. All these primary parameters were determined from the temperature dependence of the DC resistance. To cover the entire temperature range, a double-wall dipstick into the standard liquid helium storage Dewar with minimal temperature of 1.8 K was used^10^. The resistance of the tested VN films and structures determined with probing current equal to ~ 1 μA using conventional 4-point measuring circuit method. Measuring current was chosen taking into account avoidance of the sample overheating by joule heat. The value of such a current can be estimated by comparing the energy of the thermal motion of electrons (k_B_T_c_) at the expected temperature of the transition to the superconducting state, to the electron energy in the electric field of the current source (eU). This comparison allowed us to obtain the condition for measuring current I ≤ k_B_T_c_/eR, which was performed in our experiment. Figure 1a presents the temperature dependences of the resistance of the VN structures with different dimensions of sensitive area. As can be seen from the Fig. 1a, both VN structures have two pronounced superconducting transitions. The first transition at the temperature T_c1_^0.4×0.4^ ≈ 5.6 K and with ΔT_c1_^0.4×0.4^ ≈ 0.5 K for the structure 0.4 μm × 0.4 μm and T_c1_^10×10^ ≈ 6.0 K and with ΔT_c1_^10×10^ ≈ 0.1 K for the structure 10 μm × 10 μm corresponds to the transition to the superconducting state of the VN film enclosed between the arms of a spiral logarithmic antenna or contact pads. The second lower temperature transition, T_c2_^0.4×0.4^ ≈ 3.6 K with ΔT_c2_^0.4×0.4^ ≈ 0.4 K and T_c2_^10×10^ ≈ 5.2 K with ΔT_c2_^10×10^ ≈ 0.2 K designated by the transition to the superconducting state of the part of the VN film located under normal metal contact pads of the structure (arms of a spiral logarithmic antenna) and having suppressed superconductivity due to proximity effect^14^. The resistance between two transitions to the superconducting state of a structure with a small active area of 0.4 μm × 0.4 μm is higher than for structure with a size of 10 μm × 10 μm, which is due to the shape of the contact pads. In the case of a spiral antenna, the normal metal arms have several tens of squares of linear length and provide about 15 Ohms of resistance. The remaining resistance ~ 45 Ohms provides contact resistance between the film and the antenna due to the small contact area of the current flowing from a normal metal into the VN film. It should be also noted that the change of the VN film parameters within manufacture of 0.4 μm × 0.4 μm structures is more significant than structures with sizes of 10 μm × 10 μm. The values of the parameters of the initial VN film before its structuring and the fabricated VN structures are summarized in Table 1.

**Table 1:** Basic DC parameters of the initial VN films and VN structures.

Parameter	Sample #1		Sample #2	
Sample #2	VN films	VN structure	VN films	VN structure
Substrate	Si		Si	
Thickness of VN films, nm	~ 6		~ 6	
Substrate temperature at VN film deposition, K	773		700	
Sheet resistance (R_Sq_) of VN films, Ohms/□	100		98	
Dimension of the sensitive area (*L*) × (*W*), μm	–	0.4 × 0.4	–	10 × 10
Critical temperature (T_c_), K	6.0	5.6/3.6	6.2	6.0/5.2
Width of critical temperature (ΔT_c_), K	0.05	0.5/0.4	0.08	0.1/0.2
RRR = R_300_/R_20_	1.3	0.9	1.3	1.3

## Investigation of the energy relaxation time of electrons in the resistive state of VN structures using the mixing method

For coupling with radiation in the study of the energy relaxation time of the electron temperature in the VN structures and their volt-watt responsivity fabricated VN HEB was placed on an extended hyper hemispherical Si lens Fig. 1b, which were placed on a cold plate of second stage of closed cycle GM refrigeration cryostat (Sumitomo RDK-101D) with the lowest achievable temperature ~ 2.3 К. To establish temperatures, close to the temperature of the first (high-temperature) superconducting transition (T_c1_) of VN structures a resistive heater mounted on a detector holder was used. A detailed description of the mixing method used is presented, for example, in^15^.

**Figure 2:**
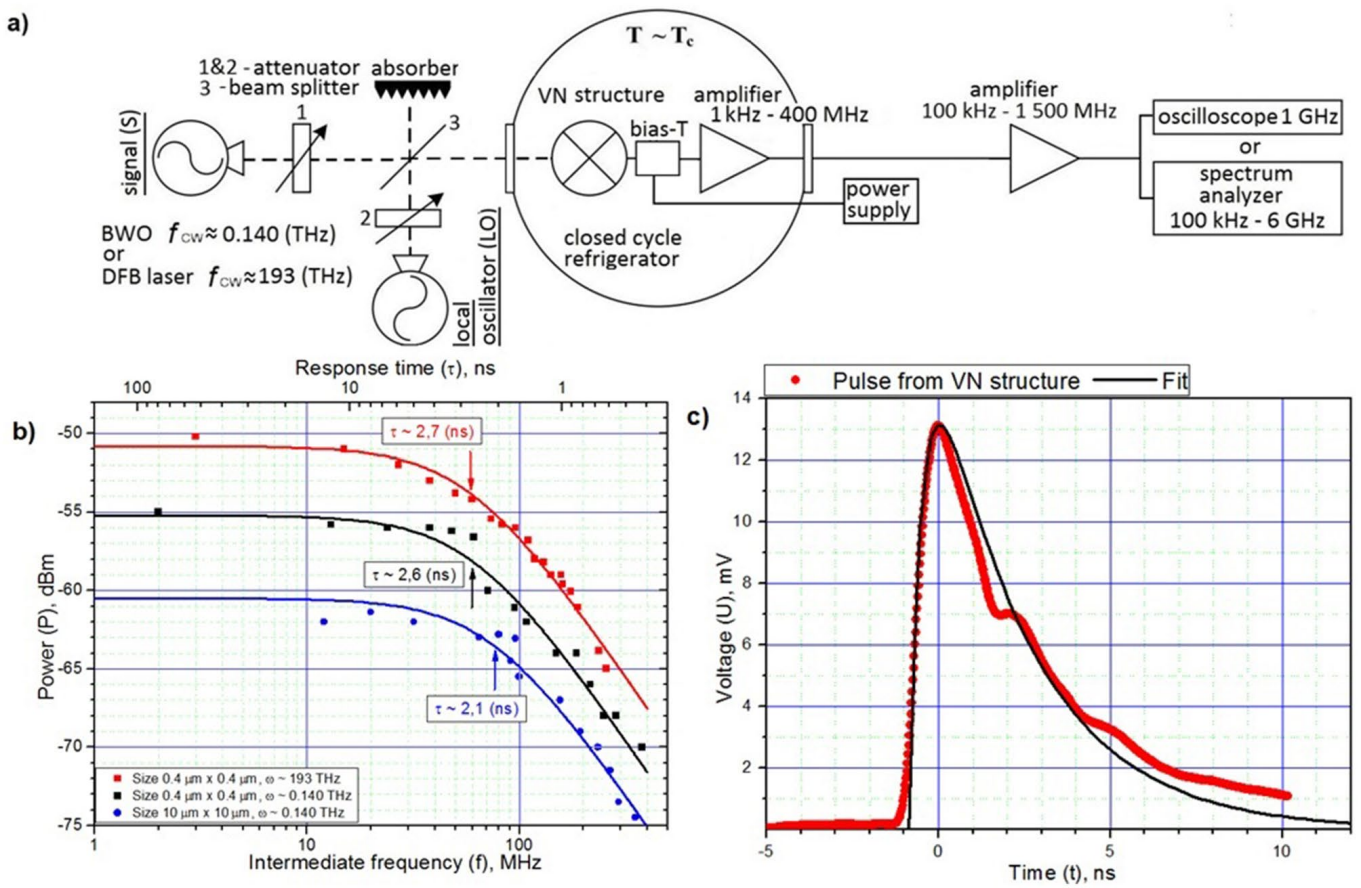
(**a**) An experimental setup for measuring the energy relaxation time of electrons in the VN structures under sub-THz or IR radiation. The energy relaxation time is measured in the mode of mixing of two radiation sources: in the sub-THz range, using backward wave oscillator (BWO) with radiation frequency near ω ≈ 0.140 THz; in the C-band IR range, using DFB lasers with a radiation frequency near ω ≈ 193 THz. (**b**) The values of the output power (P) with the VN structure depending on the intermediate frequency (f) when exposed to low-intensity high-frequency radiation with a frequency of (ω ≈ 193 THz) and (ω ≈ 0.140 THz), respectively. Black and red squares correspond to measurements on the sample with the dimensions of the sensitive region 0.4 μm × 0.4 μm; blue circles correspond to measurements on a sample with a sensitive region of 10 μm × 10 μm. All data were obtained at a temperature close to critical (in the neighborhood T_c_). Cutoff frequency (f_3dB_) and the corresponding energy relaxation time (τ) were: $f_{3dB}^{{\text{193 THz}}} \approx {\text{ 59 MHz, }}\tau_{{}}^{{\text{193 THz}}} \approx { 2}{\text{.7 ns; }}f_{3dB}^{{{0}{\text{.140 THz}}}} \approx {\text{ 61 MHz, }}\tau_{{}}^{{{0}{\text{.140 THz}}}} \approx { 2}{\text{.6 ns}}$ for sample with dimensions 0.4 μm × 0.4 μm, and $f_{3dB}^{{{0}{\text{.140 THz}}}} \approx {\text{ 76 MHz, }}\tau_{{}}^{{{0}{\text{.140 THz}}}} \approx 2.1{\text{ ns}}$ for sample with dimensions 10 μm × 10 μm, respectively. For a unifying representation of all three curves on the graph, a scale factor is introduced along the power axis. **c)** The digitized waveform (red dots) of electrical voltage pulse from the VN structure when exposed to infrared pulses. The duration of the IR pulses was < 45 ps. The black curve is an approximation curve of the form ~ A·exp(− t/τ_1_) − B·exp(− t/τ_2_), where τ_1_ = 2.8 ns, τ_2_ = 0.4 ns.

To study the energy relaxation time of electrons in the VN structures, two series of measurements were carried out using the method of mixing of two monochromatic radiation sources in the sub-THz and in the C-band IR frequency range. As sources of continuous (CW) sub-THz radiation, two high-frequency generators based on backward wave oscillator (BWO) with radiation frequency near ω ≈ 0.140 THz were selected. As sources of continuous (CW) IR radiation, two highly stabilized tunable single-mode DFB lasers with a radiation frequency ω ≈ 193 THz was used. The stability of the generation lines of both types of sources was approximate same and provided the spectral width of the signal at an intermediate frequency (IF) of (2–3) MHz. The schematic diagram of the experimental setup is presented on Fig. 2a. The IF signal was amplified by two amplification stages. The first was mounted on cryostat’s cold plate near to bolometer holder and had an operating frequency band of 1 kHz–400 MHz with a power gain of ~ 30 dB. The second amplification stage at room temperature had an operating frequency band of 100 kHz–1 000 MHz with a power gain of ~ 25 dB. In each of the experiments, the radiation frequency of one of the sources was fixed, while the frequency of the second was smoothly tuned, changing the frequency of IF signal. A spectrum analyzer with an input band of 100 kHz–6 GHz was used as a power meter for output signal at an intermediate frequency. The power level of each radiation source was maintained at a minimum level, which, on the one hand, did not lead to a significant heating of the electronic subsystem (bias current change under radiation did not exceed 1% of the bias current) and, on the other hand, provided a signal-to-noise ratio at IF of several MHz not worse than (15–20) dB. We kept the constant power of signal source at 0.140 THz while tuning its frequency by fixing the operating point of VN structure on the IV-curve.

Obtained frequency (f) dependence of the IF signal output power (P) for a sample with a sensitive area 0.4 μm × 0.4 μm are shown in Fig. 2b. The squares correspond to the experimental values of the signal power from the VN structure at different values of the intermediate frequency (f), obtained under high-frequency radiation with the frequency ω ≈ 0.140 THz (black) and ω ≈ 193 THz (red), respectively. On Fig. 2b also presented the theoretical curve of the form: P(f) = P(0) – 10Log(1 + (f/f_3dB_)^2^), where P(0) is the output power from the VN structure at zero intermediate frequency; f is the intermediate frequency (differential frequency) and f_3dB_ is cut-off frequency, corresponding to a decrease in the signal output power from the VN structure by 3 dB from its value at zero frequency P(0). The curves correspond to best fit according the least-squares method. The best approximation corresponds to the values f_3dB_ ≈ 61 MHz, for the curve ω ≈ 0.140 THz; and f_3dB_ ≈ 59 MHz, for the curve ω ≈ 193 THz.

Both curves show a good coincidence of cut-off frequencies (f_3dB_) of VN structures under high-frequency radiation of very different frequencies, which confirms the non-selectivity of the processes of electronic heating and subsequent energy relaxation in the structures of the selected topology. In addition, the process of energy relaxation of heated electrons in both cases can be attributed to a single experimentally observed time (τ), which, under the conditions of a phonon thermostat and neglecting the reverse flow of non-equilibrium phonons from the substrate to the film, it can be identified with time of the inelastic electron–phonon interaction (τ_e-ph_) and non-equilibrium phonon escape to the substrate time (τ_esc_).

as τ ≈ τ_e-ph_ + τ_esc_C_e_/C_ph_^16^, where C_e_ and C_ph_ are the heat capacities of the electron and phonon subsystems, respectively. The time constant τ of the investigated VN structures can be found from the relation τ = (2πf_3dB_)^−1^ and equals τ ≈ (2.6–2.7) ns. We also note that based on the Altshuler-Aronov formula (1)^17^, which is applicable to disordered thin films, such as the VN films, the electron–electron interaction time turns out to be equal to:

1 $$ \tau_{e - e} = \frac{\hbar }{kT}\frac{2\pi \hbar }{{e^{2} R_{Sq} }}\ln^{ - 1} \frac{\pi \hbar }{{e^{2} R_{Sq} }} $$

For the VN films, we use R_Sq_ ≈ 100 Ohms/□ and the estimate according to (1) gives τ_*e-e*_ ≈ 7.3 × 10^–11^ s at T ≈ 5.6 K, which is more than ten times less than the value we obtained in the experiment (τ). Thus, due to τ_e–e_ <  < τ in studied VN films the effect of electron heating is realized*.*

In the thin (d ≈ 6 nm) VN films used by us, the escape time of non-equilibrium phonons to the Si substrate (τ_esc_ = 4d/αu, where u is sound speed, α is acoustic match coefficient between film and substrate) cannot significantly differ from its value for typical NbN structures (50–70) ps. Also it should be note that the ratio of the C_e_/C_ph_ for thin superconducting NbN films is not great (< 1)^18,19^, and there is no any reason to assume that this ratio for VN films will be much higher. Therefore, for the structures under study τ_esc_ <  < τ_e-ph_. This fact allows us to state that the time constant τ measured by us is almost completely determined only by the electron–phonon interaction time, i.e. τ ≈ τ_e-ph_.

Also we note that the measured energy relaxation time (τ) due to the predominance of the phonon cooling channel over the diffusion channel in the general mechanism of energy relaxation of heated electrons. To confirm this, the relaxation time was measured (τ) for a VN structure having 10 μm × 10 μm the active region of the film, where the influence of the diffusion channel should be negligible. A decrease in the influence of the diffusion cooling channel should lead to an increase in the relaxation time of the electron temperature. However, measuring the dependence of the signal power at an intermediate frequency by mixing the radiation of two radiation sources with a frequency in the neighborhood ω ≈ 0.140 THz Fig. 2b showed the opposite trend. The energy relaxation time for a structure with a large distance between contacts was reduced to τ ≈ 2.1 ns. A distinctive feature of this sample was a slightly increased value of the temperature of the transition to the superconducting state (6 K versus 5.6 K), which most likely led to some shortening of the electron–phonon interaction. Numerous experimental studies of the electron–phonon interaction time (τ_e-ph_) in nitride-based compounds show that this time in a similar temperature range exhibits a dependence of the form τ ~ T^−n^, where T is the temperature at which time is measured (τ_e-ph_), n is power exponent, which typically takes the value from ~ 1.6 to ~ 3 (n = 1.6 for NbN^20^, n = 3 for TiN^6^). Then the extrapolated relaxation time value (τ) based on the expression $\tau = 2.6(ns)\left( {\frac{5.6(K)}{{6(K)}}} \right)^{n}$ gives the value from τ ~ 2.3 ns to τ ~ 2.1 ns, which is in good agreement with the measured value τ ≈ 2.1 ns. Taking this circumstance into account in combination with the fact that the length of the sensitive element of the VN structure of the small-sized structure was L = 0.4 μm and exceed the diffusion length L_diff_ ≈ (0.33–0.37) μm of electrons during the energy relaxation time (τ), which can be estimated according to the expression $L_{diff} = \sqrt {D\tau }$, where D ≈ (0.41–0.54) cm^2^/s is diffusion coefficient of electrons in the film material VN of similar thickness^12^, τ ≈ 2.6 ns is relaxation time confirms the fact that the diffusion cooling channel cannot be decisive in the general cooling mechanism of heated electrons.

## Investigation of the response of the VN structures to IR (ω ≈ 193 THz) laser pulses of picosecond duration

To study the response of the VN structures of small size to short laser pulses of the IR range ω ≈ 193 THz, we used a DFB laser in the mode of generation of radiation of short pulses with a repetition frequency of ~ 70 MHz and a duration of < 45 ps. To record pulses from the VN structure, an oscilloscope with an input band of 1 GHz was used (minimum response time ~ 0.160 ns). The peak power of laser radiation was chosen so that the response amplitude was in the linear regime for given VN structure. On Fig. 2c is presented an oscilloscope trace of an electric voltage pulse from VN structure, which occurs under the influence of IR radiation pulses. An approximation curve of form A·exp(− t/τ_1_) − B·exp(− t/τ_2_), where τ_1_ = 2.8 ns, τ_2_ = 0.4 ns. Note that the time τ_1_ agrees well with the measurements of the time constant τ by the mixing method. The time τ_2_ fully corresponds to the high-frequency cut-off of the cooled HEMT amplifier used by us (τ_ampl_ = 1/(2π400 MHz) = 0.4 ns). The observed insignificant discrepancy between the experimental and approximation curves may be due to some mismatch in the impedances of the studied VN structure and the cryogenic amplifier used, as well as the inhomogeneity of the gain.

## Investigation of volt-watt responsivity spectra of the VN structures in the frequency range ω ≈ (0.3–6) THz

**Figure 3:**
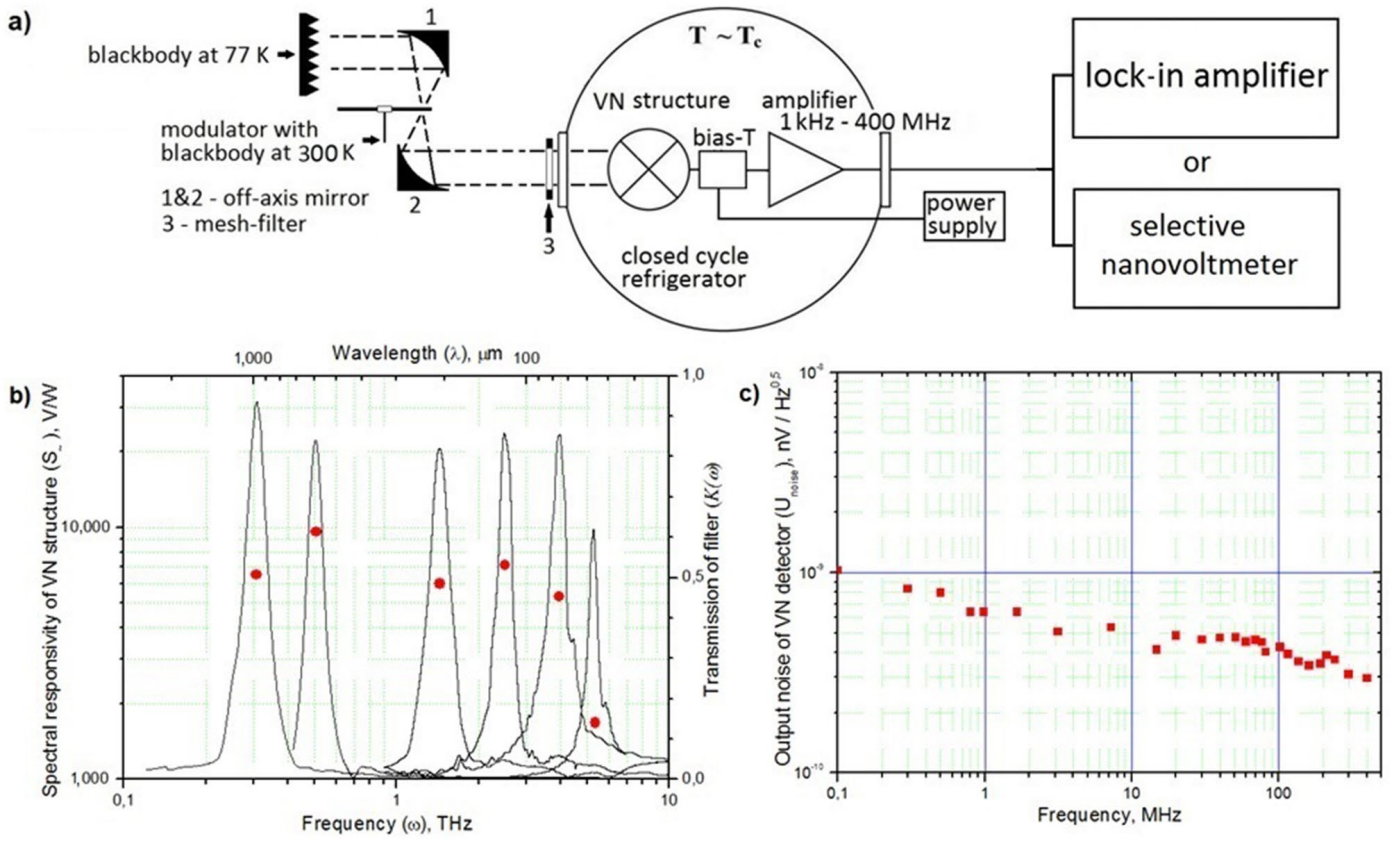
(**a**) The experimental setup for measuring the spectral sensitivity of VN structures using band-pass filters (mesh-filters) and blackbody loads in the frequency range ω ≈ (0.3–6) THz. The modulation frequency was chosen equal to ω_mod_ ≈ 3 kHz. (**b**) The dependence of the spectral sensitivity of the VN structure (red circles) and the transmittance of band-pass filters (black lines) in the frequency range ω ≈ (0.3–6) THz. The spectral sensitivity of the VN structure is recorded using the lock-in amplifier. (**c**) The dependence of the spectral density of the noise voltage of the VN detector (red squares) on modulation frequency. The spectral density of the noise voltage of the VN detector is recorded using the selective nanovoltmeter without an input radiation for the VN structures.

To study the volt-watt responsivity of the VN structures with a small active site size in the spectral range ω ≈ (0.3–6) THz, the method of amplitude modulation of THz radiation was used. The scheme of the experimental set-up used is presented on Fig. 3a. As sources of broadband THz radiation, a blackbody load was chosen at two different temperatures ~ 300 K and ~ 77 K with a set of band-pass filters (mesh-filters), installed in front of the input window of the cryostat. The amplitude modulation of THz radiation occurred at a frequency ω_mod_ ≈ 3 (kHz) using a mechanical chopper, providing 100% modulation depth with a shape close to the meander. In this experiment, only a cooled amplifier with an operating frequency band of 1 kHz–400 MHz was used. Registration of a voltage signal ($U_{\sim }^{RMS}$) arising on the VN structure at the modulation frequency ω_mod_ was carried out using a phase-sensitive synchronous detector (lock-in amplifier). The detector sensitivity ($S_{\sim }$) was calculated in accordance with the expression (2):

2 $$ S_{\sim } \approx \frac{{U_{\sim }^{RMS} }}{P} = \frac{{U_{\sim }^{RMS} }}{{\int_{0}^{\infty } {K(\omega )[h\omega \left[ {\frac{1}{{e^{{\frac{h\omega }{{k_{B} 300}}}} - 1}} - \frac{1}{{e^{{\frac{h\omega }{{k_{B} 77}}}} - 1}}} \right]]d\omega } }} $$

where $U_{\sim }^{RMS}$ is RMS signal voltage, arising on the VN structure when exposed to power P. P was calculated by full quantum–mechanical formula^21^ as integrated radiation power of blackbody loads with temperatures T_300_ ≈ 300 K and T_77_ ≈ 77 K taking into account the spectral characteristics of the transmittance of the filters. The spectral characteristics of the transmittance of the mesh-filters are recorded using a Bruker's VERTEX 70v FT-IR spectrometer.

Figure 3b represent curves of the transmission coefficient of the used band-pass filters (the black curves) and the values of the obtained volt-watt responsivity of the detector *S*_~_ (the red dots) on radiation frequency ω.

From the analysis Fig. 3b, it can be noted that for almost the entire range of the antenna’s operating range, the volt-watt responsivity of the bolometer remains almost unchanged, and only a decrease in the volt-watt sensitivity of the VN structure is observed when approaching the upper boundary of the antenna range ω > 4 THz. The decrease in sensitivity here can be explained by effect of skin depth. Narrowing bridge, the loss of the high-frequency signal in the contact areas of the spiral logarithmic antenna will increase, and mismatched bridge and antenna impedances will be introduced^22^.

## Experimental NEP of the VN detector

To estimate the noise equivalent power (NEP) of the VN detector we studied the spectral density of the noise voltage at the operating point corresponding to the maximum volt-watt responsivity S_~_ Fig. 3c. For NEP calculations used the noise voltage equal to U_noise@1MHz_ ≈ 0.6 nV/√Hz and the volt-watt sensitivity S_~_ ≈ 9 600 V/W. The minimum energy level which can be resolved by VN detector (δE) can be calculated according to the following expression^3^:

3 $$ \delta E = \frac{NEP}{{\sqrt {f_{3dB} } }} $$

where NEP is noise equivalent power, f_3dB_ is intermediate frequency bandwidth.

Table 2 summarizes the main parameters of the studied VN structures with a sensitive area of 0.4 μm × 0.4 μm, as well as the experimentally obtained value of the ultimate noise equivalent power which amounted to NEP_@1MHz_ = 6.3 × 10^–14^ W/√Hz and the minimum energy level is equal δE ≈ 8.1 × 10^–18^ J. For comparison, the same table presents the main parameters of the most common NbN HEB detectors, where NEP value is NEP = 2.5 × 10^–13^ W/√Hz^5^, which is 4 times higher than NEP of the VN HEB detectors. Note that the indicated value of NEP of the VN HEB detectors can be further reduced by creating detectors with a smaller sensitive area.

**Table 2:** Parameters of NbN and VN HEB detectors.

Parameter	NbN detector^3,5^	VN detector (this work)
	Value	
W, (m)	2 × 10^–6^	0.4 × 10^–6^
L, (m)	0.2 × 10^–6^	0.4 × 10^–6^
d, (m)	5 × 10^–9^	6 × 10^–9^
Operating temperature, (K)	9	5.6
γ, (J/cm^3^K^2^)	1.85 × 10^–4^	8.64 × 10^–4^^23^
τ, (s)	50 × 10^–12^	2.7 × 10^–9^
f_3dB_, MHz	3 200	61
NEP, (W/√Hz)	2.5 × 10^–13^	6.3 × 10^–14^ (@1 MHz)
δE, (J)	4.4 × 10^–18^	8.1 × 10^–18^

## Results

We studied the times of energy relaxation of electrons in nanostructures based on the thin VN films underheating by THz and IR bands. The measured energy relaxation time in films with a thickness of 6 nm was (2.6–2.8) ns and is determined by the electron–phonon interaction time. The volt-watt responsivity of the VN structures with the dimensions of the sensitive region 0.4 μm × 0.4 μm decreases from 9.6 kV/W at 0.5 THz to 1.5 кV/W at 6 THz due to increasing losses in the contact areas of the spiral logarithmic antenna. The obtained NEP (@1 MHz) value for VN HEB detectors was 6.3 × 10^–14^ W/√Hz, and their minimum energy level (δE) reached the value 8.1 × 10^–18^ J. The obtained values of the energy relaxation time, the volt-watt sensitivity, NEP and δE VN of the HEB detectors show their promising use and competitiveness compared to the most common NbN detectors.
